# The Intestinal Microbiota and Metabolites in the Gut-Kidney-Heart Axis of Chronic Kidney Disease

**DOI:** 10.3389/fphar.2022.837500

**Published:** 2022-03-18

**Authors:** Yinghui Huang, Wang Xin, Jiachuan Xiong, Mengying Yao, Bo Zhang, Jinghong Zhao

**Affiliations:** Department of Nephrology, The Key Laboratory for the Prevention and Treatment of Chronic Kidney Disease of Chongqing, Kidney Center of PLA, Xinqiao Hospital, Army Medical University (Third Military Medical University), Chongqing, China

**Keywords:** gut microbiota, gut-kidney-heart axis, chronic kidney disease, cardiovascular disease, intestinal dysfunction

## Abstract

Emerging evidences demonstrate the involvement of gut microbiota in the progression of chronic kidney disease (CKD) and CKD-associated complications including cardiovascular disease (CVD) and intestinal dysfunction. In this review, we discuss the interactions between the gut, kidney and heart in CKD state, and elucidate the significant role of intestinal microbiota in the gut-kidney-heart axis hypothesis for the pathophysiological mechanisms of these diseases, during which process mitochondria may serve as a potential therapeutic target. Dysregulation of this axis will lead to a vicious circle, contributing to CKD progression. Recent studies suggest novel therapies targeting gut microbiota in the gut-kidney-heart axis, including dietary intervention, probiotics, prebiotics, genetically engineered bacteria, fecal microbiota transplantation, bacterial metabolites modulation, antibiotics, conventional drugs and traditional Chinese medicine. Further, the identification of specific microbial communities and their corresponding pathophysiological metabolites and the illumination of the gut-kidney-heart axis may contribute to innovative basic research, clinical trials and therapeutic strategies against CKD progression and uremic complications in CKD patients.

## 1 Introduction

Chronic kidney disease (CKD) has become a global health problem. Currently, the prevalence of CKD has reached 10%–15% of the population worldwide (Levin et al., 2017). A large proportion of CKD patients will gradually deteriorate to end-stage renal disease (ESRD), and eventually need replacement therapy such as dialysis lifetime or kidney transplantation. Therefore, the international nephrology community has been committed to exploring new treatments to delay the progression of CKD, which is of great significance to reduce the pain and social-economic burden of CKD patients (GBD Chronic Kidney Disease Collaboration, 2020; Kalantar-Zadeh et al., 2021).

It has been well documented that the most common complication and major cause of death of CKD patients is cardiovascular diseases (CVD) (Ravid et al., 2021). The increased risk of CVD in CKD patients cannot be fully supported by traditional risk factors. Some so-called non-traditional cardiovascular risk factors, e.g. low-grade chronic inflammation, deficiency of Klotho expression and uremic toxins, have been considered as the main factors of the excess risk of CVD in CKD patients (Yang et al., 2015; Dai et al., 2017; Huang et al., 2020a; Ravid et al., 2021). Thus, the “kidney-heart” axis refers to the crosstalk between kidney and heart. Although much effort has been made to elucidate the etiology of CKD and CKD-related CVD, the efficient treatments to lower the risk of CVD and alleviate the CKD progress are still lacking in clinical practice (Ravid et al., 2021).

**FIGURE 1:**
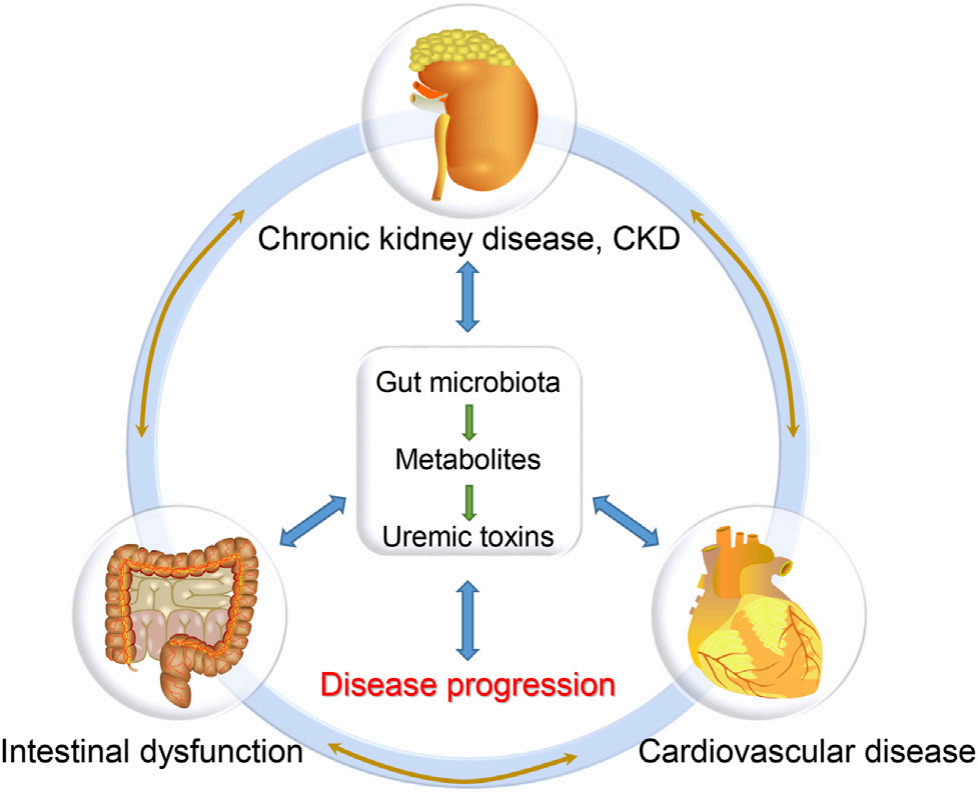
The gut-kidney-heart axis hypothesis.

During the past decades, the characteristics and functions of gut microbiota have been extensively explored in multiple disease models (Mafra et al., 2021). Many researches, including ours, have revealed the alteration of gut microbiota in CKD and CKD-related CVD patients, which might be the basis for the disease and provide novel therapeutic targets for these patients (Huang et al., 2020b). The gut is one of the important organs producing enterogenous uremic toxins, since colonic microbial metabolism substantially contributes to the production of uremic solutes (Poesen et al., 2016; Popkov et al., 2022), which are not only the result of kidney failure but also the cause of CKD progression and its complications including CVD (Aron-Wisnewsky and Clément, 2016; Caggiano et al., 2020). In the pathophysiological state of renal insufficiency, CKD patients are prone to the disorder of gastrointestinal function and intestinal microecology, which is manifested by the decrease of probiotics and the increase of pathogenic bacteria, termed as gut microbiota dysbiosis (Zhao et al., 2021; Cuevas et al., 2017). This can lead to impaired intestinal barrier and increased gut-derived toxins, which in turn accelerate the disease progression of CKD (Aron-Wisnewsky and Clément, 2016; Onal et al., 2019). Therefore, gut microbiota dysbiosis is a potential cause of CKD and CKD-related complications (Onal et al., 2019). The concept of “kidney-heart” axis can be upgraded as “gut-kidney-heart” axis (Figure 1). Under the conditions of CKD, there are reciprocal regulations among these organs (Caggiano et al., 2020). However, the precise mechanisms underlying the “gut-kidney-heart” axis remain largely unknown. In this review, we will summarize the findings of gut microbiota in CKD and CVD, and discuss their potential clinical transformations.

## 2 Gut Microbiota Under Physiological and Pathophysiological Condition

### 2.1 Physiological Effects of Gut Microbiota

Gut microbiota refer to those normal microorganisms in the human gastrointestinal (GI) tract, such as *Bifidobacteria* and *Lactobacillus*, which can synthesize a variety of vitamins necessary for human growth and development. Totally, there are more than 100 trillion bacterial cells from about 500 distinct species existing in the human GI tract (Arumugam et al., 2011). According to the cell numbers, gut microbiota can be classified as predominant and subdominant microbiota. The dominant microbiota include *Firmicutes*, *Bacteroidetes*, *Ruminococcus* and *Bifidobacterium*, which usually belong to the original flora, determining the physiological and pathophysiological significance of the host (Sabatino et al., 2015). Although gut microbiota will consume host energy and lead to diseases under certain conditions, gut microbiota do have beneficial effects on the host, which are listed below.

#### 2.1.1 Metabolic Effects

First of all, gut microbiota can decompose indigestible plant polysaccharides and resistant starch, thus facilitating the absorption of complex carbohydrates. Gut microbiota can synthesize not only a variety of vitamins, but also those necessary amino acids such as threonine and lysine (Yang et al., 2018). In healthy intestines, probiotics will continue to reproduce and vitamins can be synthesized smoothly (Zhao et al., 2021). Moreover, other metabolites of gut microbiota, such as short-chain fatty acids (SCFAs) and indole, have versatile functions (Segain et al., 2000; Yang et al., 2018). Host diet and intestinal environment significantly influence the diversity of microbiota species and their metabolites.

#### 2.1.2 Maintaining the Integrity and Function of Gi Tract

Gut microbiota contribute to the integrity and function of GI tract. In healthy subjects, the integrity of GI tract can prevent the intestinal translocation of gut microbiota, which is achieved through the intestinal barrier including the tight junctions of intestinal epithelial cells, defensive extracellular matrix and immunological mechanisms (Yang et al., 2018). Probiotics are the dominant microbiota in healthy bodies. This category of microbiota can upregulate the gene expression of mucin, the major component of mucus barrier on the apical surface of intestinal epithelial cells that can limit the binding of pathogens to epithelial cells by steric hindrance and modulate epithelial cell inflammatory signaling through their cytoplasmic domains, thus protecting the host from mucosal infection (McGuckin et al., 2011; Belzer, 2021).

#### 2.1.3 Immunological Effects

Immune responses in the gut are triggered by the interactions between the host and gut microbes (Gesualdo et al., 2021). Maturation of intestinal immune system requires the gut microbiota and microbial antigens. Dendritic cells (DCs) are professional antigen presenting cells (APCs) to capture antigens in the periphery for T cell activation. In the human gut, phagocytes are APCs, which can be distinguished on the basis of surface markers such as CD11, CCR6, CD103 and CX3CR1 (Swiatczak and Rescigno, 2012; Kim et al., 2019). These gut APCs are continuously exposed to microbial antigens, which lead to the maturation of intestinal immune system. Moreover, immunomodulation and cell differentiation of T-cells including Tregs, Th17, Th1 or Th2 cells can also be promoted by gut microbiota.

### 2.2 Pathophysiological Effects of Gut Microbiota

In the pathophysiological state, CKD and CVD patients are prone to gastrointestinal dysfunction and intestinal microecology disorder. In CKD patients, the gut microbial diversity is significantly reduced, and the microbial community is significantly different from healthy people (Nallu et al., 2017; Ren et al., 2020). A significant reduction of *Lactobacillus*, *Bifidobacterium* , *Bacteroides*, *Akkermansia*, Ruminococcaceae and *Prevotella* and proliferation of *Escherichia coli* (*E. coli*) and *Enterococcus* were detected in CKD patients, showing the reduction of probiotics and the proliferation of pathogenic bacteria (Table 1).

**TABLE 1:** The changes and functions of gut microbiota in CKD state.

Bacteria	Changes	Key functions	References
*Firmicutes*			
*Lactobacillus*	Decrease	Reverse inflammation and prevent the progression of kidney damage; protect CVD.	Yoshifuji et al. (2016), Xu et al. (2019)
*Ruminococcaceae*	Decrease	The production of butyrate has a protective effect on CKD and CVD	Jiang et al. (2017), Ahrens et al. (2021)
*Enterococcus*	Increase	Positively correlated with TMAO levels, and may serve as a biomarker of CVD.	Jiang et al, (2017), Xu et al. (2017), Liu et al. (2019)
*Actinobacteria*			
*Bifidobacterium*	Decrease	Promote colon health and produce SCFAs, delaying CKD progression.	Ranganathan et al. (2009), Vaziri et al. (2013a), Wang et al. (2020a)
*Eggerthella lenta*	Increase	Increase uremic toxins production and promote renal disease development in a CKD rat model.	Wang et al. (2020a)
*Bacteroidetes*			
*Bacteroides*	Decrease	Prevent CVD by reducing fecal LPS levels and suppressing immune response.	Vaziri et al. (2013a), Yang et al. (2018), Yoshida et al. (2020)
*Prevotella*	Decrease	Improve intestinal environment, thereby alleviating the progression of CKD and the accumulation of uremic toxins.	Mishima et al. (2015), Jiang et al. (2017), Xu et al. (2017)
*Proteobacteria*			
*Escherichia coli*	Increase	Convert tryptophan into indole by tryptophanase, and then into IS, aggravating CKD, CVD and intestinal injury.	Devlin et al. (2016), Jin et al. (2019), Huang et al. (2020b)
*Verrucomicrobia*			
*Akkermansia*	Decrease	Protect intestinal mucosa; negatively correlated with the progression of CKD and CVD.	Li et al. (2019), Wang et al. (2020b)

*Lactobacillus* is significantly reduced in CKD rats, whose intestinal barrier is impaired (Yoshifuji et al., 2016). Studies have shown that *Lactobacillus* supplementation ameliorated renal damage and intestinal injury through activation of a Toll-like receptor 2 (TLR2), a putative *Lactobacillus* receptor in the colon of CKD rats (Yoshifuji et al., 2016). In addition, *Lactobacillus* exerts a protective effect on CVD, which can alleviate myocardial dysfunction in obese mice with intermittent hypoxia by activating the nuclear factor E2-related factor 2 (Nrf2) pathway (Xu et al., 2019).

*Bifidobacterium* is a bacterial group that has been found to promote colon health, but its number is also reduced in CKD patients. Recent studies have reported that *Bifidobacterium* could slow the progression of CKD by increasing the production of microbial metabolites, such as SCFA (Ranganathan et al., 2009; Vaziri et al., 2013a). Another study showed that the probiotic *Bifidobacterium animalis* decreased the abundance of these species, reduced the levels of toxins and the severity of the disease in rats (Wang et al., 2020a).

The level of *Bacteroides* is reduced in CKD patients and rats (Yang et al., 2018), and its role in CKD needs further elaboration. However, studies have shown that *Bacteroides* can prevent CVD by reducing the levels of fecal lipopolysaccharide (LPS) and suppressing the immune response (Yoshida et al., 2020). Studies have revealed that the level of *Prevotella* is significantly reduced in CKD patients and mice with renal failure, and the restoration of *Prevotella* in the intestine helps to improve the intestinal environment, thereby inhibiting the progression of CKD and the accumulation of uremic toxins (Mishima et al., 2015; Jiang et al., 2017).

The level of *Ruminococcaceae* is reduced in CKD as well (Jiang et al., 2017). In fact, it has been found that *Ruminococcaceae* is negatively related to inflammatory bowel disease, and *Ruminococcaceae* is considered as the producer of butyrate, which is the preferred source of energy for colonic epithelial cells and plays an important role in maintaining colon health (Nallu et al., 2017). It can also retard CKD and CVD by improving lipids and blood pressure (Ahrens et al., 2021).

On the contrary, the level of *Enterococcus* is elevated in CKD patients (Jiang et al., 2017), and studies have demonstrated a positive correlation between the level of *Enterococcus* and the concentration of Trimethylamine N-Oxide (TMAO), which can serve as a potential biomarker of renal or cardiac dysfunction (Tang et al., 2015; Xu et al., 2017; Zeisel and Warrier, 2017; Liu et al., 2019). It has been reported that chronic dietary choline or TMAO directly contributed to progressive renal fibrosis and dysfunction in C57BL/6J mice (Tang et al., 2015). TMAO in the drinking water also aggravated renal inflammation and fibrosis in the high-fat diet/low-dose streptozotocin-induced diabetes rats (Fang et al., 2021). Besides, inhibition of TMAO production by using iodomethylcholine, an inhibitor of gut microbial trimethylamine, significantly attenuated the development of CKD and cardiac hypertrophy in mice (Zhang et al., 2021). These data revealed direct harmful effects of TMAO on both the kidney and heart, although no relationship was observed between serum TMAO concentrations and all-cause mortality or cardiovascular outcomes in ESRD patients requiring hemodialysis or peritoneal dialysis (Zeisel and Warrier, 2017).

*E. coli* is also significantly elevated in CKD (Devlin et al., 2016), which can convert tryptophan to indole with the catalysis of tryptophanase, and then to indoxyl sulfate, aggravating CKD, CVD and intestinal barrier injury (Jin et al., 2019; Huang et al., 2020b). It was also reported that ESRD patients showed an increase of the number of *E. coli*, leading to a significant increase of urease, uricase, indole and p-cresol forming enzymes, resulting in increased metabolites (Wong et al., 2014). In addition, *Eggerthella lenta* and *Fusobacterium nucleatum* increased the production of uremic toxins and promoted the development of renal disease in a CKD rat model (Wang et al., 2020a). Similarly, metagenomic and metabolomic studies showed a significant difference in the bacterial composition between patients with chronic heart failure and control subjects. The pathophysiological mechanisms of gut microbiota dysbiosis in CKD and CVD patients include reduced intake of dietary fibers, prolonged colonic transit time and increased amounts of protein available for proteolytic bacterial species (Nallu et al., 2017). Similarly, Karlsson et al. (2012) demonstrated that the genus *Collinsella* was enriched in patients with symptomatic atherosclerosis, while *Roseburia* and *Eubacterium* were enriched in healthy controls. Further analysis of the metagenomes revealed that the patients’ gut metagenomes were enriched in genes encoding peptidoglycan synthesis, an inflammatory component in the bacterial cell wall, and depleted in phytoene dehydrogenase, a rate-limiting enzyme involved in the metabolism of lipid-soluble anti-oxidants (Schertzer et al., 2011; Karlsson et al., 2012). These studies clearly showed that the altered microbial metabolism contributes to the pathogenesis of CKD and CVD.

## 3 Metabolites and Toxins of Gut Microbiota

Gut microbiota dysbiosis contributes to the progresses of both CKD and CVD. The best known causative links among the “gut-kidney-heart” axis are chronic inflammation and reactive oxygen species (ROS), which are induced by pathogenic bacteria or its endotoxin LPS (Aron-Wisnewsky and Clément, 2016; Li et al., 2019; Sumida and Kovesdy, 2019). Besides the indirect effects, the direct metabolites or pro-toxins originated from altered microbial metabolism have been extensively explored (Yang et al., 2018). These materials, including indoles, phenols, trimethylamine (TMA) and other metabolites, can be subsequently metabolized by the host to generate toxins (Table 2).

**TABLE 2:** The metabolites of gut microbiota.

Metabolites	Uremic toxins	Sources	Main functions	References
LPS	LPS	Bacterial cell wall	Inflammation	Yoshida et al. (2020)
Ammonia	Ammonia	Protein fermentation	Alteration of pH	Vaziri et al. (2013b)
Urea	Urea	Protein fermentation	Disruption of intestinal tight junction.	Vaziri et al. (2013b)
Phenols	PCS	Tyrosine, phenylalanine	Enhance oxidative stress, inflammation, cardiac apoptosis	Vanholder et al. (2014)
Indoles	IS, IAA	Tryptophan	Promote VC, endothelial dysfunction, cardiac hypertrophy and intestinal barrier injury	Aronov et al. (2011)
TMA	TMAO	Choline, phosphatidylcholine	Promote VC, thrombosis, platelet hyperreactivity and CVD	Liu et al. (2019)
AGEs	AGEs	Proteins and/or lipids	Induce cardiac dysfunction, vascular calcification and renal failure	Willemsen et al. (2012), Deluyker et al. (2017)
SCFAs	Butyrate	CHO fermentation	Maintain gut barrier, regulate cell growth and differentiation	Segain et al. (2000)

### 3.1 Protein Fermentation Metabolites

Colon microbes are responsible for the protein fermentation. Ammonia and urea are the products of protein catabolism, which can be converted to urea by ornithine-urea cycle (Ren et al., 2020). In colon, urea is cleaved by bacteria urease into ammonia, which in turn can alter the pH of surrounding environment and induce epithelial cell injury. Although urea is generally considered as a nontoxic retained metabolite, experiments revealed that urea caused a concentration-dependent disruption of intestinal tight junction and barrier function (Vaziri et al., 2013b).

The alpha-NH3 nitrogen (amino acids and intermediates) can be converted into phenols and indoles, which are subsequently metabolized to p-cresyl sulfate (PCS) and indoxyl sulfate (IS) or indole acetic acid (IAA). Under normal conditions, these metabolites are excreted through the kidney, but are accumulated in case of renal insufficiency (Vanholder et al., 2014). PCS and IS are prototype protein-bound uremic toxins, to which many biological and biochemical effects have been attributed (Vanholder et al., 2014). The concentrations of PCS and IS increase concomitantly with the progression of CKD because of their decreased excretion from urine (Vanholder et al., 2014).Besides, the gut microbiota dysbiosis can also result in increased production of PCS and IS. These protein binding toxins are difficult to be removed by ordinary maintenance hemodialysis (Huang et al., 2020b). Recently, a study revealed more than 30 uremic solutes in CKD patients by using high-resolution mass spectrometry, besides PCS and IS (Aronov et al., 2011). These findings suggest that uremic toxins generated from protein fermentation by gut microbiota are much complicated.

A large number of clinical and basic observations, including ours, have confirmed that PCS and IS can damage the cardiovascular system, accelerate the progression of CKD and increase the incidence of cardiovascular events in CKD patients (Vanholder et al., 2014; Yang et al., 2017; Huang et al., 2020b). Experimental studies reveal that PCS and IS can negatively affect endothelial function and repair through several mechanisms including inducing oxidative stress and inflammation.

### 3.2 Choline and Carnitine Metabolism

TMAO is an end-product of choline and carnitine metabolism (Shan et al., 2018). Carnitine is a conditionally essential metabolite that plays a critical role in cell physiology. Of note, carnitine supplementation may be associated with some clinical benefits. Choline and carnitine can be converted to TMA by gut microbiota, and then oxidized in host liver (Bennett et al., 2013; Shan et al., 2018). Like other toxins, TMAO in the circulation is excreted by the kidney, and increases in case of renal insufficiency (Bennett et al., 2013; Aron-Wisnewsky and Clément, 2016). One of the target organs is cardiovascular system, with pathophysiological effects including increasing platelet hyperreactivity and thrombosis potential, enhancing the accumulation of macrophage cholesterol and the development of atherosclerosis (Aron-Wisnewsky and Clément, 2016). In addition, dietary choline or TMAO directly contributed to renal fibrosis and dysfunction (Tang et al., 2015; Fang et al., 2021), while inhibition of TMAO production attenuated CKD development and cardiac hypertrophy in mice (Zeisel and Warrier, 2017; Zhang et al., 2021). Therefore, TMAO is recognized as a biomarker of CVD or renal dysfunction (Zeisel and Warrier, 2017).

### 3.3 Fermentation of Dietary Carbohydrates

Colon is the place where dietary carbohydrates (CHO) are fermented to SCFAs by gut microbes (Lau and Vaziri, 2019). The metabolites of SCFAs mainly include butyrate, propanoate and acetate, and their molar ratio is about 20:20:60. There are two predominant microbes in the colon, *Faecalibacterium prausnitzii* and *Eubacterium rectale*, which are responsible for producing SCFAs (Segain et al., 2000; Yang et al., 2018; Lau and Vaziri, 2019). Actually, SCFAs play an important role in maintaining energy homeostasis and gut epithelial integrity (Segain et al., 2000; Yang et al., 2018; Lau and Vaziri, 2019). One of the SCFAs is butyrate, which is a key energy substrate for the colonic epithelial cells (Zhu et al., 2021). Besides, butyrate is also known as an inhibitor of histone deacetylases, which possesses an epigenetic regulation of many genes (Segain et al., 2000; Yang et al., 2018; Lau and Vaziri, 2019). For example, inflammatory pathway was activated in colonic biopsies from patients with Crohn’s disease, but was inhibited by applying butyrate to the specimen, which was evidenced by the reduction of several pro-inflammatory cytokines (Segain et al., 2000). In addition, SCFAs can exert the anti-inflammatory effects through activating certain G protein-coupled receptors (GPRs) (Maslowski et al., 2009; Li et al., 2020). It was reported that SCFAs could increase the protein expression of GPR43, the receptor of SCFAs, in glomerular mesangial cells (GMCs) treated by high glucose and LPS, suggesting that SCFAs-induced signaling pathway might act as a therapeutic target of diabetic nephropathy (DN) (Maslowski et al., 2009; Huang et al., 2017; Li et al., 2020).

The indigestible CHO in the diet is the primary source of substrates for microbes’ fermentation. In CKD patients, CHO availability is reduced due to the dietary restrictions of potassium intake. Consistently, ESRD patients exhibited significant contraction of bacterial families possessing butyrate-forming enzymes compared with healthy subjects (Wong et al., 2014).

### 3.4 Advanced Glycation End Products

Advanced glycation end products (AGEs) are a group of proteins, lipids or nucleic acids that are glycated and oxidized after contact with reducing sugars or short-chain aldehydes (Deluyker et al., 2017). AGEs can be ingested with high temperature processed foods, but also endogenously formed as a consequence of a high dietary sugar intake (Aragno and Mastrocola, 2017; Snelson and Coughlan, 2019; Zhang et al., 2020). It has been reported that excessive sugar consumption, particularly fructose, contributes to endogenous AGEs formation, which are associated with carotid thickening, ischemic heart disease, CKD and uremic cardiomyopathy (Inagi, 2014; Aragno and Mastrocola, 2017; Gill et al., 2019). Following gastrointestinal digestion, the absorption of dietary AGEs is primarily in the forms of free-AGEs and peptide-bound AGEs (Zhang et al., 2020). Free AGEs can be absorbed by simple diffusion, while peptide-bound AGEs may be transported into intestinal cells through an intestinal peptide transporter (PEPT), PEPT1, followed by a proteolytic process into their free counterparts (Snelson and Coughlan, 2019; Zhang et al., 2020). Of note, many dietary AGEs with high molecular weight are neither absorbed in the intestine nor excreted in the feces or urine, instead, they pass through to the colon, where they may be degraded and metabolized by the colonic microbiome, although the precise mechanism remains elusive (Alamir et al., 2013; Snelson and Coughlan, 2019; Zhang et al., 2020). Recent studies observed a contraction in *Lactobacilli* both in humans and rats following a high-AGEs diet (Seiquer et al., 2014; Zhang et al., 2020). Besides, a high-AGEs diet is associated with increased intestinal permeability, decreased microbial diversity and reduced SCFAs (Snelson and Coughlan, 2019). Emerging researches have demonstrated that AGEs contribute to the development of cardiac dysfunction and renal failure mainly through cross-linking of proteins or binding to their cell surface receptor, the receptor for advanced glycation end products (RAGE), independent of diabetes (Willemsen et al., 2012; Deluyker et al., 2017). Further studies are required to elucidate the complicated network of the absorption, distribution, biotransformation and excretion of AGEs.

## 4 The “Gut-Kidney-Heart” Axis

### 4.1 Overview of the “Gut-Kidney-Heart” Axis

As renal function gradually decreases, CKD frequently damages multiple systems, including intestinal, cardiovascular and endocrine systems, among which CVD is the most common complication and the leading cause of death (over 50%), while intestinal dysfunction is also a common complication and initial symptom of CKD in many cases (Mishima et al., 2015; Schefold et al., 2016; Tuegel and Bansal, 2017; Webster et al., 2017; Huang et al., 2020a). Although we and others have confirmed the important role of the gut-kidney axis and the heart-kidney axis, few researchers have studied the gut, kidney, and heart as a whole (Huang et al., 2020a; Huang et al., 2020b). In fact, the renal, intestinal and cardiovascular systems in CKD are closely related: renal dysfunction will lead to the accumulation of numerous uremic toxins in the body, resulting in intestinal and cardiac damage; in return, intestinal dysfunction can lead to intestinal barrier destruction and microbial imbalance, promoting the production of uremic toxins and further aggravating systemic inflammation, which is also a significant inducer of renal and cardiovascular injury (Huang et al., 2020a; Huang et al., 2020b). These findings suggest that the gut, kidney and heart could interact with each other as a whole. Thus we proposed the concept of the “gut-kidney-heart” axis, whose dysfunction would lead to a vicious circle, contributing to the progression of CKD and its complications (Figure 1). However, the relevant pathogenic mechanism remains unclear, and the intervention methods need to be explored urgently.

Traditional risk factors of CVD and intestinal dysfunction also exist in CKD patients, which can hardly explain such high prevalence of the complicated diseases (Huang et al., 2020a; Huang et al., 2020b). Therefore, we focus on the effect of the gut and kidney-specific risk factors, uremic toxins and their intestinal microbiota (discussed in the part of *Pathophysiological Effects of Gut Microbiota* listed in Table 1 and Table 3).

**TABLE 3:** Potential treatments targeting microbiota in the gut–kidney-heart axis.

Potential treatments	Key findings	References
Diet intervention	**Dietary intervention:** The first-line treatment for CKD.	Lobel et al. (2020)
	**Plant-dominant low-protein diet**: Alter gut microbiome, modulate uremic toxin generation, delay CKD progression and reduce cardiovascular risks.	Conlon and Bird (2014), Gluba-Brzózka et al. (2017), Kalantar-Zadeh et al. (2020)
	**High-fiber diet:** Restore gut microbiome, reduce renal fibrosis, cardiac fibrosis, and left ventricular hypertrophy via inhibiting Egr1.	Kieffer et al. (2016), Marques et al. (2017)
	**High sulfur amino acid-containing diet**: Modulate the indole and IS levels by sulfide inhibition of TnaA, ameliorate kidney function in CKD mice.	Lobel et al. (2020)
	**Low phosphate diet**: Alleviate mitochondrial injury, vascular calcification, cardiac hypertrophy and failure.	Liu et al. (2018)
	**Ketogenic diet**: Protect obesity-associated CVD, improve renal function, increase beneficial gut microbiota; might aggravate renal dysfunction.	Ma et al. (2018), Olson et al. (2018), Bruci et al. (2020), Dowis and Banga (2021), Jia et al. (2021), Zhang (2021), Rojas-Morales et al. (2022)
Probiotics	***Lactobacillus acidophilus*** ATCC 4356: Regulate oxidative stress and inflammation, and reduce atherosclerosis.	Chen et al. (2013)
	***Lactobacillus acidophilus***: Reduce serum dimethylamine and nitrosodimethylamine, and improve ESRD.	Yoshifuji et al. (2016)
	**Lebenin**: Inhibit uremic toxins, restore microbiota of uremic patients.	Sabatino et al. (2015)
	***L. casei* Zhang**: Ameliorate AKI and CKD progression by increasing the levels of SCFAs and niacinamide.	Zhu et al. (2021)
	***Bifidobacterium*:** inhibit inflammation, protect intestinal barrier, and alleviate CKD progression.	Rui-Zhi et al. (2020)
	***Streptococcus thermophilus, Lactobacillus acidophilus and Bifidobacterium longum***: Reduce BUN levels, improve kidney function in CKD patients.	Ranganathan et al. (2009), Ranganathan et al. (2010)
Prebiotics	**p-inulin**: Reduce inflammation, serum PCS and IS, improve metabolic function.	Cani et al. (2007), Meijers et al. (2010)
	**Acarbose**: Reduce serum p-cresol concentration.	Evenepoel et al. (2006)
	**Fiber**: Reduce inflammation and mortality in CKD patients.	Krishnamurthy et al. (2012)
	**Resistant starch**: Ameliorate IS, PCS and CKD in rats.	Kieffer et al. (2016)
Genetically engineered bacteria	S-sulfhydration or mutation of *E. coli* TnaA reduces its activity, thus alleviating serum IS levels and kidney injury.	Lobel et al. (2020)
	Deleting *Bacteroi*des TnaA eliminates the production of indole and controls IS levels.	Devlin et al. (2016)
Fecal microbiota transplantation	FMT from CKD patients: Induce serum uremic toxins, renal fibrosis and oxidative stress in mice.	Barba et al. (2020)
	FMT from AKI mice: Aggravate the kidney injury in I/R-induced AKI mice.	Zhu et al. (2021)
	FMT from healthy mice: Improve gut microbiota disturbance and decrease PCS accumulation in CKD mice.	Caggiano et al. (2020)
Bacterial metabolite modulation	**Indole absorbents**: **AST-120.** Decrease serum IS and AGEs, delay the initiation of hemodialysis, restore intestinal barrier and reduce inflammation in CKD models.	Ueda et al. (2006), Ueda et al. (2007), Yamaguchi et al. (2017), Huang et al. (2020b)
	**TMA inhibitor: DMB**. Inhibit microbial TMA formation, plasma TMAO levels, endogenous macrophage foam cell formation and atherosclerotic lesion development.	Wang et al. (2015)
	**AGE formation inhibitors (synthetic compounds and natural products)**: Block sugar attachment to proteins, attenuate glycoxidation, break down formed AGE crosslinks	Liu M. et al. (2020)
	**RAGE antibody or gene knockout**: Alleviate renal injury and development of nephropathy.	Flyvbjerg et al. (2004)
	**Small molecule inhibitors of RAGE** (RAGE229): Reduce diabetic complications by inhibiting the interaction between the cytoplasmic tail of RAGE and Diaphanous-1.	Manigrasso et al. (2021)
Antibiotics	**Vancomycin**: Decreased IS and PCS in ESRD patients.	Nazzal et al. (2017)
	**Antibiotics**: Improve kidney injury by preventing inflammatory response.	Furusawa et al. (2013)
	IR-induce AKI and CKD models in germ-free mice show more severe renal damage.	Jang et al. (2009), Mishima et al. (2017)
Conventional drugs	**Lubiprostone**: Ameliorate CKD progression and uremic toxins, restore *Lactobacillaceae* family and *Prevotella* genus.	Mishima et al. (2015)
	**Metformin**: Increase *Lactobacillus* and *Akkermansia*, improve atherosclerosis and gut barrier integrity.	Lee and Ko (2014), Li et al. (2016), de La Cuesta-Zuluaga et al. (2017), Caggiano et al. (2020)
	**Acarbose**: Increase *Lactobacillus* and *Bifidobacterium*, deplete *Bacteroides*, regulate bile acid metabolism	Gu et al. (2017)
	**SGLT2i**: Reduce uremic toxins, modulate *Firmicutes* to *Bacteroidetes* ratio, increase SCFA-forming bacteria.	Lee et al. (2018), Mishima et al. (2018), Caggiano et al. (2020)
Traditional Chinese medicine	**Jian-Pi-Yi-Shen decoction**: Improve renal function via modulating Clostridium_XIVb in CKD rats.	Zheng et al. (2020)
	**Qing-Re-Xiao-Zheng formula**: Protecte renal function via regulating gut microbiota dysbiosis and inhibiting inflammation in DKD rats.	Gao et al. (2021)
	**Shenyan Kangfu tablet**: Increase *Firmicutes* and decrease *Bacteroidetes* in diabetic mice.	Chen et al. (2021)
	**Mahuang decoction**: Ameliorate kidney impairment, restore microbiota dysbiosis.	Ming et al. (2021)

### 4.2 Substances Mediated the Crosstalk of the “Gut-Kidney-Heart” Axis

#### 4.2.1 Cardiorenal Syndrome or Kidney-Heart Axis

As much as 30% of CKD patients have coexisting heart failure (HF), while 40–50% of HF patients suffer from chronic renal dysfunction (Mishima et al., 2015; Schefold et al., 2016; Tuegel and Bansal, 2017; Webster et al., 2017; Huang et al., 2020a). Traditional risk factors of CVD are insufficient to explain the high prevalence of HF in CKD patients (Go et al., 2004; Yang et al., 2015; Chuppa et al., 2018). Therefore, increasing attention was paid to CKD-inherent risk factors, i.e. gut microbiota (Table 1) and their metabolites (Table 2), which are derived from gut microbiota and accumulated in the body during disease progression (Huang et al., 2020a).

As shown in Table 2, multiple gut microbiota-derived uremic toxins, including IS, TMAO and AGEs, were involved in the progresses of both CKD and CVD (Wang et al., 2015; Hudson and Lippman, 2018; Huang et al., 2020a; Huang et al., 2020b). For example, we demonstrated that IS resulted in cardiac hypertrophy and CKD-associated thrombosis via enhancing oxidative stress in mice (Yang et al., 2015; Yang et al., 2017). IS predicts CVD and renal function deterioration (Lin et al., 2012).

In addition, after screening a series of uremic toxins, we identified phosphate as a potential therapeutic target, since a high-phosphate diet-induced cardiac hypertrophy and heart failure through regulating interferon regulatory factor 1 (IRF1)-peroxisome proliferator-activated receptor gamma coactivator 1 alpha (PGC1α) axis, thus affecting mitochondrial energy metabolism remodeling in CKD mice (Huang et al., 2020a). Besides, we and others have previously shown that phosphate levels were negatively associated with left ventricular ejection fraction (LVEF) and estimated glomerular filtration rate (eGFR) (Huang et al., 2020a). High phosphate could also induce vascular calcification via the downregulation of peroxisome proliferator-activated receptor-gamma (PPARγ) in CKD mice or the activation of toll-like receptor 4 (TLR4)/nuclear factor-κB (NF-κB) signaling in vascular smooth muscle cells (VSMCs) (Zhang et al., 2017; Liu et al., 2018).

#### 4.2.2 Gut-Kidney Axis

Emerging evidences, including ours, have revealed that the intestinal epithelial barrier is impaired both structurally and functionally in CKD patients and mice, while modulating the microbiome can preserve kidney function (Meijers et al., 2018; Caggiano et al., 2020; Huang et al., 2020b). Further studies verified the involvement of uremic toxins in intestinal barrier injury (Vaziri et al., 2013b; Xie et al., 2014). As reported, advanced oxidation protein products (AOPPs) induced intestinal epithelial cell death through a redox-dependent pathway (Xie et al., 2014). An *in vitro* experiment observed disruption of the intestinal barrier by urea (Vaziri et al., 2013b). We demonstrated for the first time that IS, a uremic toxin derived from intestinal *E. coli*, induced intestinal barrier injury through regulating IRF1-dynamin related protein 1 (DRP1) pathway, thereby blocking the mitochondrial autophagy flux and inducing mitochondrial injury, which is the common damaged subcellular organelle in renal, intestinal and cardiovascular dysfunction, as well as a potential therapeutic target in CKD state (Huang et al., 2020a; Huang et al., 2020b; Srivastava et al., 2020; Bi et al., 2021). Despite the preliminary research on the damaging effect of the uremic toxin on gut injury, the interaction between uremic toxin and intestinal flora and its mechanisms are far from clear.

**FIGURE 2:**
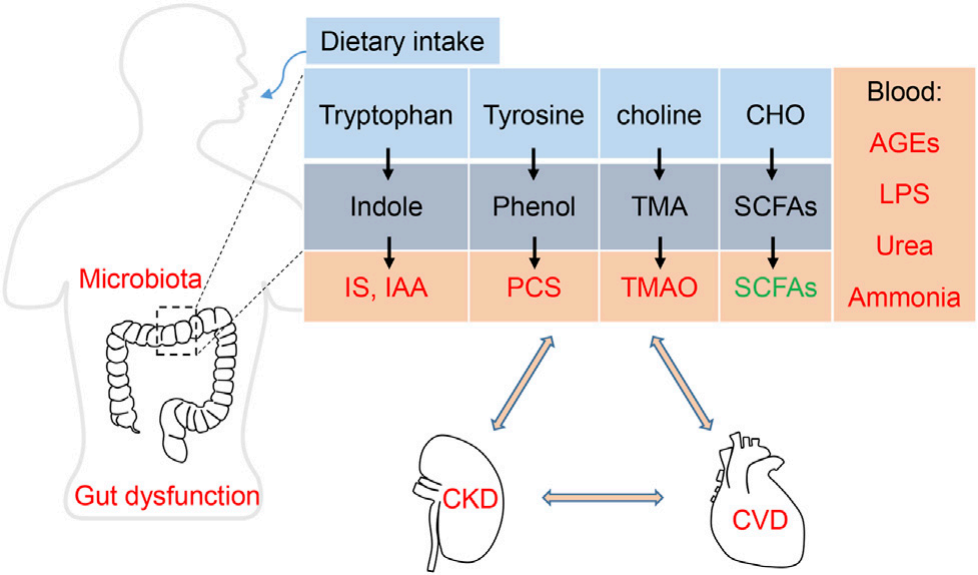
Uremic toxins in the gut-kidney-heart axis.

Based on the close relationship among the three organs, we can refer them as the “kidney-gut-heart” axis (Figure 2). Future studies revealing the key pathogenic mechanism of the kidney-gut-heart axis and prospectively exploring novel therapeutic targets and strategies for clinical prevention and treatment hold clinical significance for improving the prognosis of CKD patients.

## 5 Potential Therapeutic Strategies Targeting Gut Microbiota and its Metabolites Against Chronic Kidney Disease Progression in the Gut-Kidney-Heart Axis

Considering the close relationship between gut microbiota and the gut-kidney-heart axis, the role of protein-binding toxins that are metabolized by the intestinal flora and may in turn damage the intestinal, renal and cardiovascular system and are difficult to remove by routine dialysis, deserves special attention in CKD (Masereeuw and Verhaar, 2020). Uremic toxins, mainly derived from dietary metabolites, are not only the result of kidney failure but also promote the progress of CKD via inducing various pathogenic signals (Inagi et al., 2014). Therefore, potential therapies aimed at modulating the microbiota dysbiosis and lowering their pathogenic metabolites could be promising therapeutic strategies in the gut-kidney-heart axis.

### 5.1 Dietary Intervention

Dietary intervention serves as the first-line treatment for CKD, due to its role in improving CKD outcomes and preventing or delaying dialysis initiation (Kalantar-Zadeh et al., 2020; Lobel et al., 2020). Modifying dietary protein intake has been identified as a clinical strategy in the management of CKD patients for decades (Lobel et al., 2020). Many diet-microbiome studies have focused on the effects of dietary fiber, protein and phosphates (Conlon and Bird, 2014; Kalantar-Zadeh et al., 2020; Lobel et al., 2020).

#### 5.1.1 Plant-Dominant Low-Protein Diet

High-protein diets not only result in higher CVD risks but also faster CKD progression because of increased intraglomerular pressure, glomerular hyperfiltration and the production of nitrogenous end-products (Conlon and Bird, 2014; Kalantar-Zadeh et al., 2020). In contrast, a plant-dominant, fiber-rich, low-protein diet may lead to favorable alterations in the gut microbiome, which can modulate the generation of uremic toxins and slow the progression of CKD and CVD (Kalantar-Zadeh et al., 2020). It has also been reported that plant proteins may exert beneficial effects on blood pressure, proteinuria, glomerular filtration rate and renal tissue damage compared to animal proteins (Gluba-Brzózka et al., 2017). Thus, a plant-based diet may prevent the development of uremic complications including CVD (Gluba-Brzózka et al., 2017).

#### 5.1.2 High-Fiber Diet

A high-fiber diet can restore intestinal microbiome, and plasma, cecal and urine metabolome, as well as retarding the progression of CKD (Vaziri et al., 2014; Kieffer et al., 2016). These effects were mediated by restoration of intestinal epithelial tight junction, attenuation of the oxidative stress, inflammation and fibrosis (Vaziri et al., 2014). Further, a high-fiber diet increased the abundance of SCFAs-producing bacteria (Marques et al., 2017). Both fiber and acetate alleviated gut dysbiosis and increased the levels of *Bacteroides acidifaciens*, leading to reduced renal fibrosis, cardiac fibrosis, and left ventricular hypertrophy possibly through downregulating cardiac and renal early growth response protein 1 (Egr1), a master cardiovascular regulator involved in cardiac hypertrophy and cardiorenal fibrosis (Marques et al., 2017).

#### 5.1.3 Low-Phosphate Diet

We have demonstrated that a high-phosphate diet induced heart failure through regulating myocardial energy metabolism remodeling in CKD mice (Huang et al., 2020a). Further, each 20% increase in dietary phosphate intake was associated with an estimated 1.1 g greater left ventricular mass in a community-based study (Yamamoto et al., 2013). As reported, hyperphosphatemia is an independent risk factor for mortality in dialysis patients and in patients with stages 3–4 CKD (Kestenbaum et al., 2005). Of note, phosphate binders can ameliorate cardiac hypertrophy and dysfunction in CKD mice, and improve the survival rate in CKD patients (Tonelli et al., 2010; Maizel et al., 2013). Therefore, phosphate consumption should be restricted for CKD patients.

#### 5.1.4 High Sulfur Amino Acid Diet

Dietary sulfur-containing amino acid intake has also been reported to ameliorate CKD progression in patients and disease models (Lobel et al., 2020). Lobel et al. (2020) found that a high sulfur amino acid-containing diet modulated the production of indole and IS via sulfide inhibition of tryptophanase (TnaA), thus abrogating uremic toxicity and ameliorating kidney function in CKD mice. Therefore, high sulfur amino acid diet can improve gut microbiota through posttranslational modification without altering the composition of microbial community (Lobel et al., 2020).

#### 5.1.5 Ketogenic Diet

A ketogenic diet constitutes a high fat and low carbohydrate diet that stimulates endogenous ketone body production (Zhang, 2021). A ketogenic diet is generally protective to obesity-associated CVD, while remaining contradictory to diabetes and other metabolic disorder-related CVDs (Dowis and Banga, 2021; Zhang, 2021). It can also attenuate acute and chronic ischemic kidney injury mainly through reducing oxidative stress and inflammation (Rojas-Morales et al., 2022). Further, a prospective observational study showed that a 3-month very low-calorie ketogenic diet leads to not only weight loss but also improvement of renal function in obese patients (Bruci et al., 2020; Dowis and Banga, 2021). Mechanistic studies showed that the increased gut microbiota, *Akkermansia* and *Parabacteriodes* mediated the anti-seizure effects of ketogenic diets in mouse models (Olson et al., 2018). Another research revealed that ketogenic dietary interventions enhanced brain vascular function possibly through increasing beneficial gut microbiota, such as *Akkermansia*, *Muciniphila* and *Lactobacillus* (Ma et al., 2018). However, it should also be noted that ketogenic diets could lead to metabolic disorders and aggravate renal dysfunction in spontaneously hypertensive rats (Jia et al., 2021). Therefore, further studies and randomized controlled trials are warranted to evaluate the therapeutic possibilities of ketogenic dietary interventions (Dowis and Banga, 2021; Zhang, 2021).

### 5.2 Probiotics

Probiotics are defined as “living microorganisms” composed of live bacteria, such as *Bifidobacteria*, *Lactobacillus* and *Streptococci* (Rastall et al., 2005). In CKD patients, oral treatment with *Streptococcus thermophilus*, *Lactobacillus acidophilus* and *Bifidobacterium longum* can slow the progression of kidney disease (Ranganathan et al., 2009; Ranganathan et al., 2010). Oral *Lactobacillus* acidophilus for hemodialysis patients significantly reduced serum dimethylamine, a potential uremic toxin (Yoshifuji et al., 2016). In another study, treatment with *Lactobacillus acidophilus* ATCC-4356 reduced the burden of atherosclerosis in apolipoprotein e (ApoE)^−/−^ mice (Chen et al., 2013). In uremic patients undergoing hemodialysis, oral antibiotic-resistant lactic acid bacteria preparations, *Lebenin*, can normalize the composition of the intestinal microbiota and inhibit the accumulation of uremic toxins, including phenol, p-cresol and indicant (Sabatino et al., 2015). The beneficial effect of *Lactobacillus* may be the result of the influence of this bacterium on the permeability and immune status of the intestinal epithelium.

A recent study demonstrated that oral probiotic *Lactobacillus casei* (*L. casei* Zhang) corrected intestinal microbial imbalance, alleviated kidney damage, and delayed the progression to CKD through increasing the levels of SCFA and nicotinamide in mice (Zhu et al., 2021). In patients with stage 3–5 CKD, oral administration of *L. casei* Zhang also slowed down the decline of renal function (Zhu et al., 2021). Various approaches based on the use of probiotics, prebiotics and synbiotics can adjust the intestinal microbiota and intestinal barrier structure/function, thereby alleviating CKD and its complications: CVD and intestinal dysfunction (Sabatino et al., 2015; Cuevas et al., 2017).

### 5.3 Prebiotics

Prebiotics are non-digestible food ingredients that have beneficial effects by selectively stimulating the growth or activity of bacteria in the colon. Common prebiotics contains inulin, fructooligosaccharides, galactooligosaccharides, soybean oligosaccharides, xylo-oligosaccharides and pyrodextrins (Sabatino et al., 2015). Evidence showed that prebiotic oligofructose-enriched inulin (p-inulin) could promote the growth of *bifidobacteria* species, inhibit inflammation and improve metabolic function (Cani et al., 2007). Another report found that oral intake of p-inulin reduced the serum concentrations of PCS and IS in hemodialysis patients (Meijers et al., 2010). A number of clinical trials and experimental studies on CKD have also shown that taking prebiotics, probiotics and synbiotics can reduce uremic toxins and inflammatory mediators, as well as improving colonic epithelial tight junctions (Vaziri et al., 2014; Ramezani et al., 2016).

A diet with high levels of resistant starch, a prebiotic, is beneficial to change the characteristics of the gut microbiota and cecum, serum and urine metabolites, such as IS, in adenine-induced CKD rats (Kieffer et al., 2016). In addition, high dietary fiber intake is associated with a low risk of inflammation and mortality in CKD patients (Krishnamurthy et al., 2012).

### 5.4 Genetically Engineered Bacteria

It was reported that S-sulfhydration or mutation of *E. coli* TnaA reduced its activity as measured by indole production from tryptophan both *in vitro* and in bacterial cultures, thus alleviating serum indoxyl sulfate levels and kidney injury (Lobel et al., 2020). In addition, another study identified a widely distributed family of TnaA in the gut commensal *Bacteroides* and found that deleting TnaA eliminates the production of indole *in vitro* (Devlin et al., 2016). Further altering the status or abundance of the *Bacteroides* TnaA also modulated IS levels *in vivo* (Devlin et al., 2016). These results suggest a new strategy for treating CKD by genetically modulating the microbiome and the downstream products.

### 5.5 Fecal Microbiota Transplantation

Fecal microbiota transplantation (FMT) is an emerging therapy involving the transfer of stool from healthy donors into the GI tract of diseased recipients (Mamic et al., 2021). FMT has already been approved for administration in the treatment of recurrent *Clostridium difficile* infection (McDonald et al., 2018), which is the only application reported so far. It has also been highlighted that FMT could restore the dysbiosis to promote host homeostasis, including the control of intestinal inflammation through secreting IL-10 (Cammarota et al., 2017; Burrello et al., 2018).

Recently, several studies demonstrated that the implantation of fecal microbiota from CKD patients into renal injured germ-free mice or antibiotic-treated rats could induce higher production of uremic toxins, renal fibrosis and oxidative stress (Kelly, 2013; Kootte et al., 2017). Transplantation of stool samples obtained from donor AKI mice also aggravated the kidney injury in ischemia-reperfusion (I/R)-induced AKI mice (Zhu et al., 2021). Reversely, FMT treatment from healthy mice significantly improved gut microbiota disturbance and decreased PCS accumulation in CKD mice (Caggiano et al., 2020).

Further studies, especially clinical trials, are required to investigate the feces amount, the timing and the efficiency of FMT, which may serve as a therapeutic option in CKD patients. Of note, strict and comprehensive donor screening protocols remain essential to avoid adverse infectious events, since two immunocompromised FMT recipients suffered from *E. coli* bacteremia originating from the gut microbiota of the donor in two independent clinical trials (DeFilipp et al., 2019). Further identification of the benefits and risks of FMT across different populations are also indispensable. Despite these challenges addressed above, FMT may still be a feasible option in the future.

### 5.6 Bacterial Metabolites Modulation

#### 5.6.1 Uremic Toxin Absorbent (AST-120)

IS and PCS belong to protein-bound uremic toxins, which can hardly be efficiently removed by conventional dialysis (Masereeuw and Verhaar, 2020). Hence, therapies aimed at reducing the production of these bacterial metabolites could be promising therapeutic strategies. We and others have demonstrated that AST-120 partially restored intestinal epithelial tight junction and reduced oxidative stress and inflammation in CKD models (Huang et al., 2020b). A carbonaceous oral adsorbent AST-120 can bind many low-molecular-weight compounds, including indole, the precursor of indoxyl sulfate, in the intestines and prevent IS production (Yamaguchi et al., 2017). AST-120 can also bind AGEs to decrease serum levels of AGEs in patients with CKD (Ueda et al., 2006). In addition, administration of AST-120 can delay the initiation of hemodialysis in CKD patients (Ueda et al., 2007). AST-120 administration can not only prevent renal and cardiac fibrosis in the 5/6-subtotal nephrectomy CKD mouse model through reducing serum IS levels (Fujii et al., 2009; Lekawanvijit et al., 2012), but also improves cardiac dysfunction in ischemia/reperfusion-induced AKI mice via the suppression of apoptosis and nuclear factor kappa-light-chain-enhancer of activated B (NF-κB) signaling (Shen et al., 2021). These studies further supported the gut-kidney-heart axis.

However, several multicenter randomized trials have not supported the benefit of AST-120 for CKD patients (Akizawa et al., 2009; Schulman et al., 2015). Recently, a systematic review found that oral administration of AST-120 reduced serum levels of IS but did not prevent disease progression or reduce all-cause mortality of CKD patients (Chen et al., 2019), which highlights the complicated pathophysiology of CKD and the requirement of removing more than one single uremic toxin (Masereeuw and Verhaar, 2020).

#### 5.6.2 Trimethylamine Inhibitor

3, 3-dimethyl-1-butanol (DMB) is a structural analog of choline, which can reduce plasma TMAO levels through inhibiting microbial TMA formation, the first step in TMAO generation in mice fed a high-choline diet (Wang et al., 2015). Further, DMB inhibited endogenous macrophage foam cell formation and atherosclerotic lesion development in ApoE^−/−^ mice (Wang et al., 2015), suggesting DMB as a potential therapeutic approach for the treatment of cardiometabolic diseases.

#### 5.6.3 AGE Formation Inhibitors

Both synthetic compounds and natural products have been evaluated as inhibitors against the formation of AGEs (Liu M. et al., 2020). The underlying mechanisms of AGEs inhibitors were mainly through blocking sugar attachment to proteins, attenuating glycoxidation via scavenging the intermediates and breaking down the formed AGEs’ crosslinks (Liu M. et al., 2020). Most synthetic compounds, such as aminoguanidine and pyridoxamine prevented the formation of AGEs through scavenging reactive carbonyls and radicals formed during glycation, or blocking the formation of intermediate Amadori products at the late stage of glycation (Liu M. et al., 2020). Some synthetic inhibitors, including aspirin and diclofenac, exerted their effects by interfering with the initial attachment between reducing sugars and amino groups, thus inhibiting the further formation of Schiff base and AGEs (Liu M. et al., 2020).

The natural plant extracts eriodictyol and naringenin could inhibit the formation of AGEs via stabilizing the structure of bovine serum albumin (Liu J. et al., 2020). The resveratrol glucosides inhibited AGEs formation by their antioxidant and methylglyoxal-trapping capacity (Liu M. et al., 2020). Considering the side effects of synthetic molecules in clinical trials, natural products are more promising to be developed as clinical AGEs inhibitors (Nagai et al., 2014; Liu M. et al., 2020).

#### 5.6.4 Receptor for Advanced Glycation End-Products Inhibitors

The receptor for advanced glycation end-products (RAGE) is a multiligand pattern recognition receptor implicated in the pathogenesis of multiple inflammatory disease states including CKD, atherosclerosis and CVD (Hudson and Lippman, 2018). Reducing glycation stress via modulation of the AGE-RAGE signaling may represent a promising approach for preventing CKD progression (Inagi, 2016).

RAGE and AGEs are upregulated in podocyte cells of the glomerulus in mice and patients with diabetic nephropathy (Tanji et al., 2000). Blocking RAGE activation with either RAGE antibody or gene knockout can alleviate renal injury (Wendt et al., 2003). As reported, a neutralizing RAGE antibody reduced basement membrane thickness, mesangial volume, urinary albumin excretion and serum creatinine levels in db/db mice (Flyvbjerg et al., 2004). In addition, RAGE gene knockout protected against the development of atherosclerosis in both diabetic and nondiabetic ApoE-null mice (Harja et al., 2008; Soro-Paavonen et al., 2008).

Recent studies suggested TTP488 as an orally bioavailable small-molecule inhibitor of RAGE in diabetic complications and CVD (Hudson and Lippman, 2018). Very recently, Manigrasso et al. developed a small molecule inhibitor of RAGE, RAGE229, which attenuated the complications of diabetes-associated kidney disease in mouse models by inhibiting the interactions between the cytoplasmic tail of RAGE and Diaphanous-1, thus completely inhibiting the intracellular RAGE signaling (Manigrasso et al., 2021). Further clinical trials are needed to test whether these compounds are effective therapeutics.

### 5.7 Antibiotics

Recent studies suggested that removing the gut microbiota with oral antibiotics, vancomycin, can significantly decrease the plasma concentration of two gut-derived uremic solutes, IS and PCS, in ESRD patients (Nazzal et al., 2017). Antibiotic administration also improved kidney I/R injury by preventing inflammatory response (Furusawa et al., 2013).

However, the I/R-induce AKI and CKD models in germ-free mice showed more severe renal damage than in mice with commensal intestinal microbiota (Jang et al., 2009; Mishima et al., 2017), making the conclusions contradictory. Of note, the administration of large-spectrum antibiotics is not recommended as a long-term therapy because of disturbing intestinal microbiota (Knoop et al., 2016) and enriching antibiotic-resistant bacteria (Baym et al., 2016). Therefore, further studies and clinical trials are required to explore the efficacy of antibiotics in patients.

### 5.8 Conventional Drugs

Lubiprostone is a synthetic derivative of prostaglandin activating chloride channel, frequently used in the treatment of constipation. In a rat model of CKD, oral administration of lubiprostone decreased the plasma level of microbiome-derived uremic toxins, such as IS and hippurate, and attenuated tubulointerstitial damage, renal fibrosis and inflammation probably through restoring *Lactobacillaceae* family and *Prevotella* genus (Mishima et al., 2015).

Accumulated studies demonstrated the microbiota-modulating effects of antidiabetic drugs. Metformin could increase *Lactobacillus* and *Akkermansia* in diabetic mice and patients, which can also improve atherosclerosis and gut barrier integrity (Lee and Ko, 2014; Li et al., 2016; de La Cuesta-Zuluaga et al., 2017; Caggiano et al., 2020).

The α-glucosidase inhibitors, such as acarbose, increased the relative abundances of *Lactobacillus* and *Bifidobacterium* and depleted *Bacteroides*, thereby regulating the relative abundance of microbial genes involved in bile acid metabolism in type 2 diabetes (T2D) patients (Gu et al., 2017).

In fact, multiple studies revealed an increased ratio of *Firmicutes*/*Bacteroidetes* (F/B) in diabetes (Caggiano et al., 2020). It has been demonstrated that sodium glucose cotransporter 2 inhibitors (SGLT2i) could reduce uremic toxins, improve vascular dysfunction and modulate the gut microbiota, including the decrease of F/B ratio and the increase of colonic SCFAs-forming bacteria, in diabetic mouse models (Lee et al., 2018; Mishima et al., 2018; Caggiano et al., 2020). Though contradicting with other studies, it cannot be ignored that Shenyan Kangfu tablet (SYKFT), a prescription of traditional Chinese medicine for treating CKD, was reported to enhance the relative abundance of *Firmicutes* and reduce *Bacteroidetes* in diabetic mice with diabetic kidney disease (Chen et al., 2021). In-depth experiments are indispensable to distinct the accompanying phenomena or causal efficacy between microbiota and conventional drugs-induced effects (Magne et al., 2020).

### 5.9 Traditional Chinese Medicine

Very recently, increasing evidences showed the beneficial effects of traditional Chinese medicine (TCM) in CKD treatment through modulating microbiota. Jian-Pi-Yi-Shen (JPYS) decoction not only improved renal function but also restored the blood reticulocyte counting and serum calcium level possibly through modulating *Corynebacterium*, *Coprococcus*, and *Clostridium_XIVb*, which may serve as candidate microbiome targets for further studies on their roles in medicating the drug efficacy of TCM in 5/6 nephrectomized CKD rats (Zheng et al., 2020). Qing-Re-Xiao-Zheng formula protected the renal function via regulating gut microbiota dysbiosis, reducing the levels of gut-derived LPS and inhibiting inflammatory responses in rats with diabetic kidney disease (DKD) (Gao et al., 2021). Another report demonstrated that herbal formula granule prescription Mahuang decoction ameliorated kidney functional and structural impairment in CKD rats, which were associated with the restoration of the impaired richness and diversity of intestinal microflora and the disrupted microbial community (Ming et al., 2021). However, further mechanism studies and intervention experiments, such as FMT and germ-free mice, are still in urgent need to prove the role of gut microbiota in TCM-mediated effects.

### 5.10 Future Therapeutic Interventions Based on Sequencing Techniques

The emergence of high-throughput bacterial DNA sequencing techniques, including 16S ribosomal DNA (rDNA) microbial profiling and shotgun metagenomics, has facilitated better understanding of human gut microbiota composition and illumination of their pathophysiological roles in human diseases (Ventura et al., 2009; Marchesi et al., 2016; Ticinesi et al., 2018). It has been demonstrated that whole genome shotgun sequencing has multiple advantages compared with the 16S amplicon method, including deeper characterization of the microbial complexity, enhanced detection of bacterial species, increased detection of diversity, identification of more taxa and increased prediction of genes (Ranjan et al., 2016; Laudadio et al., 2018; Liu Y. et al., 2020). Several studies combined 16S and metagenomic sequencing to analyze the gut microbiota composition and function of stone formers to gain a more comprehensive understanding of the changes in the gut-kidney axis in kidney stones (Ranjan et al., 2016; Ticinesi et al., 2018; Liu Y. et al., 2020). Further, the 16S rDNA sequencing or shotgun metagenomic data in combined with the profiles of transcriptome, proteomics and metabolomics of gut microbial community, may contribute to novel therapeutic targets against the diseases of the host.

## 6 Conclusion and Future Perspectives

The gut microbiota are a single molecule, which may produce important molecules or substances, playing a vital role in multiple diseases, especially including CKD, CVD and intestinal dysfunction. In this review, we propose an expansion of the cardiorenal syndrome and gut-kidney axis, and consider these three organs as a whole, gut-kidney-heart axis, whose dysfunction would lead to a vicious circle, contributing to the progression of CKD and its complications. Further, recent studies suggest potential therapeutic strategies targeting gut microbiota against CKD progression in the gut-kidney-heart axis, including dietary intervention, probiotics, prebiotics, genetically engineered bacteria, fecal microbiota transplantation, bacterial metabolites modulation, antibiotics, conventional drugs and traditional Chinese medicine. We and others have also demonstrated that mitochondria may serve as a potential therapeutic target, although there is still a lack of clinical interventions.

It should be noted that the current studies are preliminary, and mechanistic experiments and clinical trials are urgently required to further advance the understanding of the efficiency and safety of these potential therapeutic strategies. Besides, it’s still a significant challenge in the field of gut microbiota to translate metagenomic findings into biologically relevant mechanisms. This might be achieved through the isolation of bacterial strains, identification of the corresponding pathophysiologic metabolites, analysis of their response to specific stimulating factors and the linking of these findings to disease-specific biomarkers or pathophysiologic parameters. Therefore, targeting gut microbiota could yield novel therapies against CKD progression.

Despite these limitations, the experimental and clinical data available suggest that modulation of the intestinal microbiota in the gut-kidney-heart axis could be promising therapeutic strategies for CKD patients, especially for those with complications of CVD and intestinal dysfunction.
